# Influence of Three-Dimensional Printing Parameters on Compressive Properties and Surface Smoothness of Polylactic Acid Specimens

**DOI:** 10.3390/polym15183827

**Published:** 2023-09-19

**Authors:** Hamed Bakhtiari, Mostafa Nikzad, Majid Tolouei-Rad

**Affiliations:** 1 School of Engineering, Edith Cowan University, Joondalup, WA 6027, Australia; 2 Department of Mechanical and Product Design Engineering, School of Engineering, Swinburne University of Technology, Hawthorn, VIC 3122, Australia; mnikzad@swin.edu.au

**Keywords:** additive manufacturing, process optimization, compressive properties, surface smoothness, fused filament fabrication

## Abstract

While the mechanical performance of fused filament fabrication (FFF) parts has been extensively studied in terms of the tensile and bending strength, limited research accounts for their compressive performance. This study investigates the effect of four process parameters (layer height, extrusion width, nozzle temperature, and printing speed) on the compressive properties and surface smoothness of FFF parts made of Polylactic Acid (PLA). The orthogonal Taguchi method was employed for designing the experiments. The surface roughness and compressive properties of the specimens were then measured and optimized using the analysis of variance (ANOVA). A microscopic analysis was also performed to identify the failure mechanism under static compression. The results indicated that the layer height had the most significant influence on all studied properties, followed by the print speed in the case of compressive modulus, hysteresis loss, and residual strain; extrusion width in the case of compressive strength and specific strength; and nozzle temperature in the case of toughness and failure strain. The optimal design for both high compressive properties and surface smoothness were determined as a 0.05 mm layer height, 0.65 mm extrusion width, 205 °C nozzle temperature, and 70 mm/s print speed. The main failure mechanism observed by SEM analysis was delamination between layers, occurring at highly stressed points near the stitch line of the PLA prints.

## 1. Introduction

**Figure 1:**
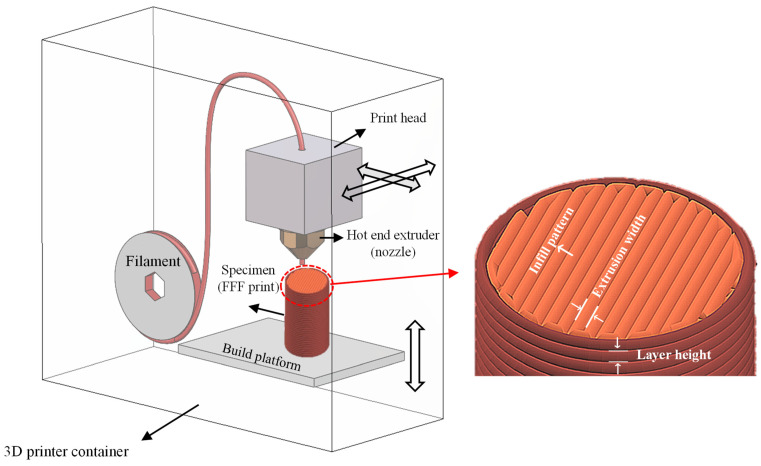
Schematic illustration of the FFF process, and some geometrical features of an FFF print.

Material extrusion (ME) refers to additive manufacturing techniques that involve the layer-by-layer extrusion of molten or semi-liquid materials to create plastic, metal, or composite parts [1]. FFF (fused filament fabrication), also known as FDM (Fused Deposition Modeling), is an ME technique that utilizes a heated nozzle to melt and deposit materials onto a build platform. FFF-printed parts have found extensive applications as biomedical scaffolds [2], composite structures [3], shape memory components [3], and functional prototypes in various industries, such as medical, automotive, and electronics [4,5,6]. A schematic of an FFF machine and the resulting printed part is depicted in Figure 1. The print head can move freely in the XY plane while the height is adjusted by the movement of the build platform in the Z direction. In the deposition stage, the new layer is bonded to the successive layer through the fusion mechanism [7].

The compressive strength and surface finish in fused filament fabrication (FFF) parts play critical roles in various demanding applications. Notably, FFF finds application in metal forming and molding tools [8,9,10], jigs and fixtures [11,12], full-scale molds for wind turbine blades [13], packaging products [14,15], bone scaffolds [2], and medical implants [16], where compressive strength is essential to withstand compressive stresses. The surface finish is equally crucial in such applications. For instance, in 3D printing die tools for sheet metal drawing, the surface roughness influences the friction between the die and sheet metal, affecting the quality of the drawn metal. Similarly, in the automotive industry, strict dimensional requirements necessitate a smooth surface finish for body panels to ensure a proper fit and alignment, highlighting the significance of surface quality in precise part manufacturing.

The mechanical strength of the FFF parts (also referred to as “print” in this paper) is greatly influenced by the process and geometrical parameters [17,18]. Several studies in the literature have investigated the effect of process parameters on the mechanical performance of FFF parts. Cojocaru et al. [19] conducted a review study on the effect of process parameters on the mechanical properties of Polylactic Acid (PLA) prints, concluding that thinner layers resulted in smaller internal cavities and improved surface quality, layer bonding, and mechanical properties. It was also shown that mechanical properties can be improved when a grid infill pattern and horizontal build direction were used in the 3D printing of specimens. However, high printing speeds can negatively impact surface quality due to incomplete solidification, and low or high nozzle temperatures can cause incomplete melting or material flow issues. Process parameters such as the nozzle temperature and print speed impact the temperature profile (heating and cooling cycles), subsequently affecting the bonding strength between layers [20,21].

The effect of the process and geometrical parameters on the mechanical behavior of prints can vary based on the type of loading. For example, research has shown that decreasing the raster angle improves the tensile strength of a print [22,23,24,25] but decreases its compressive strength [25,26]. This is while no clear correlation has been found between the raster angle and fatigue life of prints [27]. This discrepancy stems from the different failure modes under different loads. In tension, raster breaking is the main failure mode, so the highest load-carrying capacity occurs when the extruded filaments are aligned with the loading direction. On the other hand, parts are more likely to buckle under compressive force, so having the filaments arranged perpendicular to the applied load leads to the best compressive strength results [26]. Gordelier et al. [28] found that the optimal tensile strength in ABS and PLA prints was attained with a raster angle of 0°, minimum layer height, and horizontal build direction. Bakhtiari et al. [27] studied the influence of printing parameters on the fatigue performance of FFF prints. They found that cross-over infill patterns like the grid and honeycomb outperformed unidirectional raster patterns in tensile fatigue strength. Notably, the 0° and 45° raster angles exhibited the highest fatigue strength, while the printing speed (25–70 mm/s) had a comparatively weaker influence, inversely affecting the tensile fatigue life.

Although the tensile and bending strength of FFF prints has been extensively studied in the literature, there is a limited account of their compressive performance. Gabor et al. [29] investigated the compressive strength of PLA prints at various build orientations (0°, 15°, 30°, 45°, 60°, and 90°) and found that a 0° orientation yielded the highest strength. In another study [30], the build orientation was found to have the biggest influence on the compressive strength of ABS prints followed by the layer height, raster angle, and air gap, while the extrusion width exhibited an insignificant impact. Compressive strength was directly correlated with layer height and extrusion width but inversely with build orientation and air gap. Dave et al. [31] examined the compressive strength of PLA prints at different layer heights (0.1–0.3 mm), infill densities (60–80%), and print speeds (30–50 mm/min). The findings indicated that the compressive strength peaked at a layer height of 0.2 mm before declining. They also demonstrated that compressive strength directly correlated with infill density due to the reduced interlayer cavities and greater material volume at higher infill densities, which enhanced the mechanical support. Print speed had a minor effect, showing a slight increase up to 40 mm/min.

**Figure 2:**
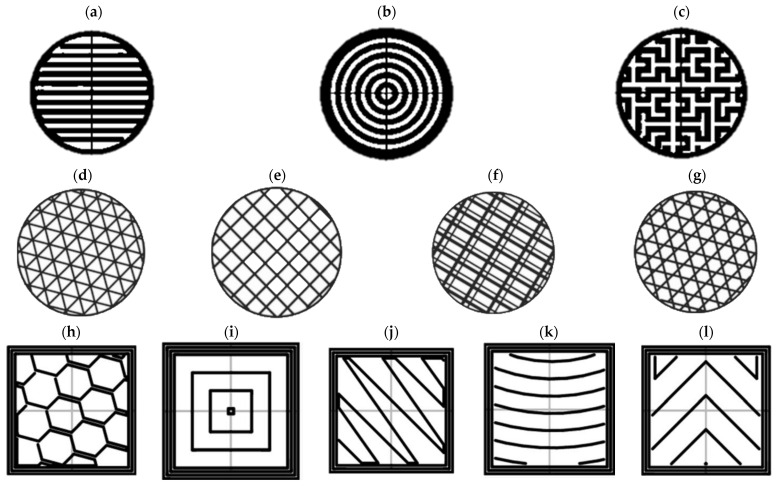
Infill patterns investigated in compression tests: (**a**) rectilinear, (**b**) concentric-circle, (**c**) Hilbert curve, (**d**) triangle, (**e**) grid, (**f**) quarter cubic, (**g**) tri-hexagon, (**h**) honeycomb, (**i**) concentric-rectangle, (**j**) line, (**k**) Archimedean, and (**l**) Octagram spiral (**a**–**c**) reproduced with permission from Springer [32], (**d**–**g**) reproduced with permission from Elsevier [33], (**h**–**l**) reproduced with permission from Springer [34]).

Infill patterns significantly impact print strength by influencing the contact area and layer bonding. Figure 2 depicts various infill patterns studied for the compressive behavior of FFF prints. Prajapati et al. [32] examined PLA prints with varying infill patterns (rectilinear, concentric, and Hilbert curve) with the highest compression strength achieved using the rectilinear pattern. In [33], PLA prints with a triangular infill design demonstrated a superior compressive strength than the grid, quarter cubic, and tri-hexagon patterns due to having a larger contact area between the layers. Similarly, the Hilbert curve pattern exhibited a superior compressive strength than the other designs (honeycomb, line, rectilinear, Archimedean curve, and Octagram spiral) due to the strong bonding between the rasters and layers [34].

Material choice significantly affects the compressive strength in FFF prints due to its rheological and microstructural characteristics. ABS and nylon, for example, exhibit increased compressive strength as the layer height decreases [35,36]. Reducing the layer height leads to an increased shear rate within the extruding polymer, causing a viscosity drop in materials with shear-thinning properties [37], such as ABS and nylon [38,39]. This, in turn, enhances the layer fusion and overall strength. In contrast, PEEK is a non-shear-thinning polymer, resulting in the layer height having minimal influence on the compressive strength [40]. In addition, ABS is an amorphous polymer, while PEEK is semi-crystalline. The microstructural differences between these materials can also affect their fusion and recrystallization behavior during the 3D printing process. Other studies have looked at the effect of the printing speed on the strength of PLA [31] and PEEK [41] prints, with little to no significant changes observed. Table 1 presents the results found in the literature regarding the influence of the FFF process parameters on the compressive properties and surface quality of different prints.

The surface smoothness of 3D-printed PLA parts is also influenced by various printing parameters as summarized in Table 1. The surface roughness is a measure of surface smoothness, meaning that lower surface roughness is an indicator of a smoother surface. While the raster angle [42] has been shown to have a negligible effect on the surface finish in some studies, the layer height [42,43,44], extrusion width [42,45,46], and print speed [46] were found to enhance the surface finish when reduced. Higher infill densities also contribute to smoother surfaces [34,47]. The impact of the air gap and nozzle temperature varies in different studies, with some suggesting them influential [48,49] and others finding them negligible [42,43,46]. Similarly, the build orientation had varying effects on surface roughness, exhibiting either a decreasing trend [43] or an oscillating pattern [44].

**Table 1:** Influence of FFF process parameters on the surface and compressive properties of FFF prints.

		Influence on Mechanical and Surface Properties	
		Compressive Properties	Surface Quality
	**Nozzle temperature**	High nozzle temperature can result in hardness increase and thermos-oxidative degradation of polymers at the same time, leading to increase in strength [35]	Changing the nozzle temperature shows no significant effect on the surface roughness of FFF prints [43,46] The surface roughness decreases with increasing nozzle temperature [49]
**3D printing parameters**	**Raster angle**	Rasters perpendicular to the loading direction yield the highest compressive strength [26,30]	The highest surface finish is attained at zero raster angle; however, its effect is negligible [42]
	**Infill pattern**	Compressive strength of rectilinear pattern > concentric and Hilbert curve pattern [32] Compressive strength and specific strength of hexagonal pattern > linear > triangular pattern [50] Compressive strength of 0/90° and 45/−45° infill patterns are almost equal in horizontal prints made of Ultem 9085 [51] Compressive strength of grid pattern > triangle > tri-hexagon > quarter cubic [33] Compressive strength of Hilbert curve pattern (121.35 MPa) > rectilinear > line > Archimedean > honeycomb > Octagram [34]	Rectilinear pattern exhibits the lowest surface roughness as compared to Hilbert curve and line patterns [34]
	**Infill density**	Compressive strength and modulus increase by increasing the infill density [31,32,34,50,52]	Surface roughness decreases significantly by increasing the infill density [47]
	**Layer height**	Within 0.1–0.3 mm range, 0.2 mm layer height produces the highest compressive strength in PLA print [31] Compressive strength of ABS prints exhibits both inverse [35] and direct relationship [30] with the layer height Compressive modulus increases with decreasing layer height Layer height shows little impact on compressive strength of PEEK prints [40]	Decreasing the layer height improves the surface quality [42,43,49] Reducing the layer height results in a decrease in surface roughness in 20 and 45° build orientations, but it increases at 70° build orientation [44]
	**Printing speed**	No significant effect [31,41]	Decreasing the print speed enhances the surface finish of PLA prints [46]
	**Build orientation**	Compressive strength decreases by increasing the build orientation [29,30,32,51] Horizontal build direction exhibits 15–40% higher compressive strength than that of vertical build direction in Ultem 9085 prints [51] Compressive strength of ABS prints is not impacted by build direction [53]	Increasing the build orientation angle decreases the surface roughness in [43] and shows an increasing–decreasing trend in [44]
	**Air gap**	Compressive strength exhibits inverse relationship with the air gap [30]	The lowest surface finish is attained at zero air gap [48] Negative air gap degrades the surface quality [42] Changing the air gap shows no significant effect on the surface roughness of FFF prints [43]
	**Extrusion width**	Extrusion width exhibits insignificant impact on compressive strength of ABS prints [30]	The best surface finish is attained at the highest extrusion width [42,45] Decreasing the extrusion width enhances the surface roughness [46] in the extrusion width direction Changing the extrusion width shows no significant effect on the surface roughness of FFF prints [43]

While the tensile and flexural strengths of FFF prints have received extensive attention in the literature, their compressive performance has been relatively underexplored. The aim of this study was to examine the effect of the layer height, extrusion width, nozzle temperature, and printing speed on the surface roughness and compressive properties of PLA specimens. The Taguchi method was employed to design experiments and optimize the printing parameters for maximum compressive strength and surface smoothness. Physical and mechanical tests were conducted to determine the density, surface quality, and compressive properties of the specimens. Furthermore, a microscopic analysis was carried out to identify the underlying failure mechanism under static loading. The findings of this study provide further insights into optimizing the 3D printing process for applications requiring strong compressive properties and high surface quality.

## 2. Materials and Methods

### 2.1. Sample Preparation

**Figure 3:**
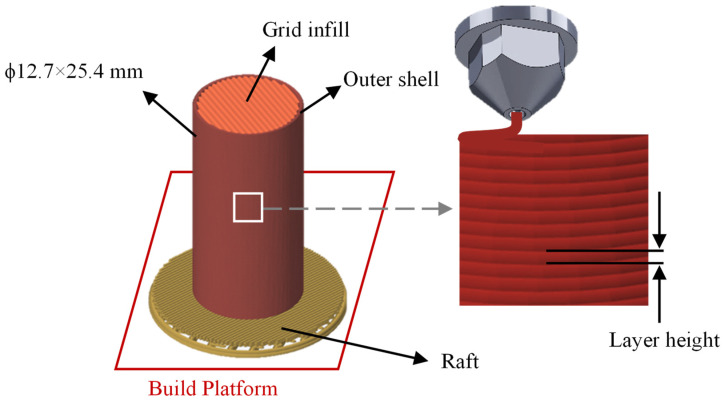
Schematic illustration of a sample printed in this study.

In the present study, PLA filament (X3D pro PLA), with a diameter of 1.75 mm, was used to fabricate samples. Cylindrical samples (ϕ12.7 × 25.4 mm) were designed and layered using ideaMaker 4.4.0 software and then 3D printed using a Raise3D Pro3 Plus printer. Figure 3 shows a typical printed sample. To help improve the adhesion of the sample to the build plate, a raft platform was added to the base of each specimen and the build plate was pre-heated to a 60 °C. 0°/45° grid pattern with the infill density of 100%, and one solid shell layer was used to build the specimen.

### 2.2. Design of Experiments

The layer height, extrusion width, nozzle temperature, and print speed were considered as the variable parameters. The design of the experiments was performed by employing the orthogonal array Taguchi method in Minitab 21.4.0.0 software. Table 2 shows the variable with different levels (low, medium, and high) used as well as the response parameters and their definitions. 

The configuration of the designed experiments is shown in Table 3. The minimum and maximum level of the factors were selected according to the range given by the manufacturer as well as some trial and error. The relative density, surface roughness, compressive strength, compressive modulus, specific strength, failure strain, hysteresis loss, and residual strain were set as the response parameters.

### 2.3. Density Measurement

**Figure 4:**
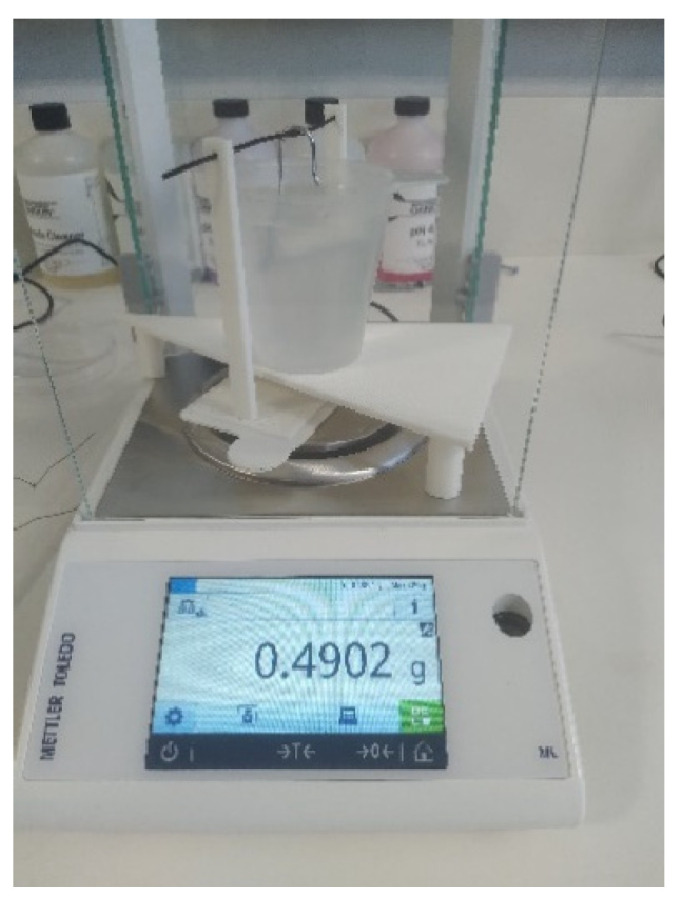
Digital balance for density measurements.

The density measurements were conducted using the Archimedes method according to ASTM D792 [54]. A digital balance with a precision of 0.0001 gr was used for weighing the samples in air and in submerged states, and distilled water was used for submerging the samples (Figure 4). Each sample was weighed in air and then submerged in distilled water using a sinker. The test was carried out in the standard laboratory temperature and the water temperature of 28 °C. The apparent density of each sample was calculated according to Equation (1).

(1) $$\rho_{sample}(g/{cm}^{3})=\frac{m_{a}}{\left(m_{a}-m_{w}\right)}\times \rho_{w}$$

where $\rho_{w}$ is the density of water at the test temperature (0.996 gr/cm^3^), and $m_{a}$ and $m_{b}$ are the apparent mass of the sample in air and in water, respectively. To calculate the relative density of each sample, its apparent density was divided by the density of the filament (1.26 gr/cm^3^) which represents the density of a fully dense sample (Equation (2)).

(2) $$\rho_{rel}=\frac{\rho_{sample}}{\rho_{Filament}}\times 100$$

To make sure about the validity of the results, three specimens were tested for each group, making a total of 27 experiments. Then, the arithmetic average of the measured densities in each group was reported.

Following the density measurement, the samples underwent drying via a fan-forced flow of air at a temperature of 35 °C for a duration of 1 h, utilizing a sunbeam electronic dehydrator machine. Subsequently, the mass of each sample was determined and compared to its initial mass to confirm the absence of any water within the samples. The findings revealed a maximum mass change of 0.0005 g among the 27 specimens tested, indicating the complete evaporation of all the water present within the samples.

### 2.4. Surface Roughness

**Figure 5:**
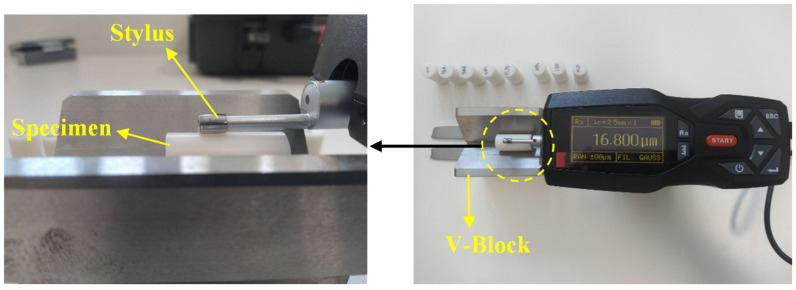
Setup for surface roughness measurement.

Having a high-quality surface finish is of paramount importance for improving both the functionality and appearance of 3D-printed parts. In addition, it can also help reduce costs by minimizing the amount of post-processing needed and speeding up the overall prototyping process. In the present study, the TMR200 surface roughness tester, manufactured by PCWI Co., was employed for the surface measurements. The instrument featured a 5μm tip, which was employed for all surface roughness measurements. The cutoff length was set at 2.5 mm as recommended by the manufacturer, ensuring coverage of the entire length of at least five consecutive layers in the 3D-printed samples. A stainless-steel V-block was used to mount the specimens in the lateral direction. Figure 5 shows the configuration of the surface roughness setup.

**Figure 6:**
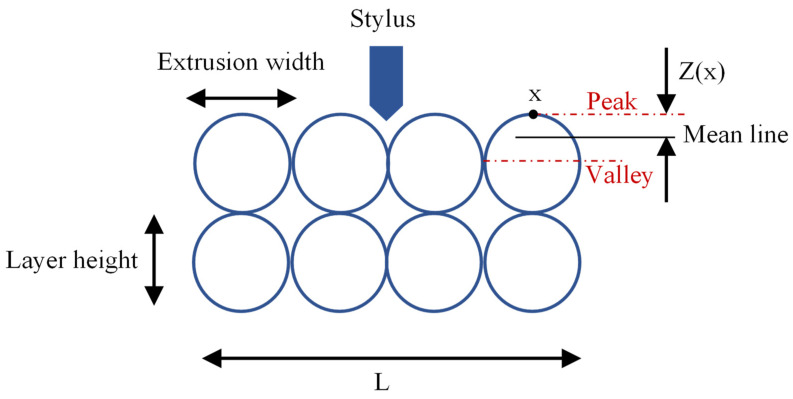
FFF prints configuration and surface roughness measurement.

The roughness tester was calibrated before testing using a calibration block provided by the manufacturer. The stylus was brought into contact with the surface of interest while maintaining consistent pressure. The stylus was then moved along the sample’s length in a straight line, and its displacement was measured and converted into an electrical signal. The processed signal was then used to calculate the Ra using Equation (3).

(3) $$R_{a}=\frac{1}{L}\int_{0}^{l}\left|Z(x)\right|dx$$

where *R_a_* is the average surface roughness, *L* is the sampling length along the *x* direction, and *Z* (*x*) is the height of the surface profile relative to the mean line at the distance *x*. Figure 6 depicts the configuration of the FFF prints and the surface roughness measurement.

Each test was repeated three times at different locations, and the average of three recorded *R_a_* was reported as the surface roughness.

### 2.5. Compression and Hysteresis Compression

The quasi-static compression tests were conducted using an Instron 8801 device. The device was equipped with a double-acting servo hydraulic actuator, capable of applying forces up to ±100 kN, within a 150 mm stroke range, and at loading rates between 0.1 mm·min^−1^ and 240 mm·s^−1^. All the compression tests were carried out in accordance with the ASTM D695 standard [55], which covers the determination of the compressive properties of rigid plastics. The compression tests were conducted at a temperature of 23 ± 2 °C and relative humidity of ~6%. The compression speed was set at 1.3 mm/min and each test was repeated five times to guarantee the statistical accuracy and reliability of the results.

**Figure 7:**
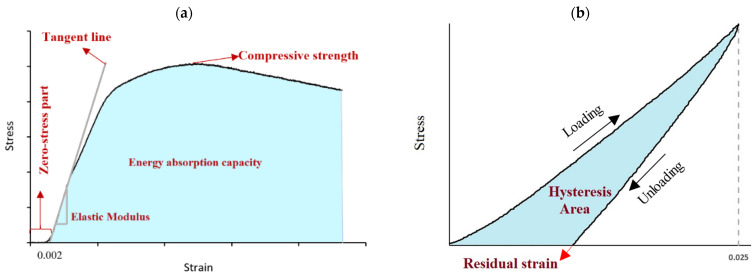
Typical stress–strain curve of a specimen under (**a**) compression and (**b**) hysteresis compression.

Prior to testing, the diameter and height of the fabricated specimens were measured to the nearest 0.01 mm at several points for the stress and strain measurements, respectively. The minimum diameter was used to calculate the cross-sectional area. The load–deformation curve was recorded at a data recording frequency of 20 Hz, and the resulting stress–strain curve was obtained by dividing the load and deformation values to the minimum cross-sectional area and initial sample’s length, respectively. The zero-stress portions of the stress–strain curves were then ditched from the graphs, as shown in Figure 7a. All the samples were subjected to both a uniaxial destructive compression test and hysteresis (loading–unloading) compression, and each test was repeated three times for statistical validation. Figure 7 illustrates a typical stress–strain curve of a specimen under compression and hysteresis compression.

The compressive modulus was calculated by drawing a tangent line to the initial linear portion of the stress–strain curve, selecting a point on this line, and dividing the compressive stress represented by this point by the corresponding strain. The compressive strength and specific strength of each sample were then obtained by identifying the maximum compressive stress and dividing it by the sample’s density, respectively. The specific strength of the FFF prints is studied in this paper because it is an important mechanical property that indicates the strength-to-weight ratio of the printed parts, which is crucial in many engineering applications where lightweight and high-strength components are required. Finally, the toughness (energy absorption capacity) of each sample was acquired by measuring the area under the stress–strain curve up to the failure point in Figure 7a.

The area between the loading and unloading curves in Figure 7b indicates the amount of energy that is lost due to the material’s viscoelastic nature. In fact, when plastics are deformed, a phase shift between the stresses and strains occurs, resulting in the hysteresis loop [56]. The energy that is dissipated in one full cycle is represented by the area of this hysteresis loop. Like the compression test, the loading and unloading in the hysteresis compression were performed at the rate of 1.3 mm/min. The samples were kept in the unloading state for 10 min for static recovery. To make sure that the loading does not surpass the elastic region, the yield strain of each sample was determined from the compression tests, and hysteresis loading was performed up to the 0.025 strain, which is in the elastic region of all the samples. After loading achieved the pre-defined stroke, the unloading stage followed until the complete release of the load. The remaining deformation after unloading was measured and reported as the residual strain. Also, the area confined between the loading and unloading curves was calculated as the hysteresis loss.

### 2.6. Scanning Electron Microscopy (SEM)

Scanning electron microscopy was carried out using a JEOL benchtop SEM machine to investigate the fracture characteristics of the specimens. Three-dimensional printed samples were mounted on a holder and imaging was conducted around the fractured areas with an accelerating voltage of 15 kV.

## 3. Results and Discussion

Table 4 provides the results obtained from the physical and mechanical experiments for the 3D-printed samples.

### 3.1. Analysis of Variance (ANOVA)

The analysis of variance (ANOVA) determines the statistical significance of various factors on different responses. Each response is fitted using the linear regression as Equation (4).

(4) $$y=\beta_{0}+\left(\beta_{k}\times x_{k}\right)$$

where y, β_k_, and x_k_ represent the response, coefficients, and factors, respectively. Minitab 21.4.0.0 software was used in this study for the statistical analyses. Depending on the regression data, the transformation of responses might be necessary to meet the assumptions of normality and homogeneity of variance [57]. In the present study, the Box–Cox equation (y′ = y *^λ^)* was utilized to apply transformation to the surface roughness, compressive strength, compressive modulus, specific strength, toughness, failure strain, hysteresis loss, and residual strain using λ values of 0.5, 3, 3, 4, 4, 7, −1, and 0.5, respectively. Table 5 provides the results obtained by the ANOVA for each response, including the developed regression equations. The significance of each factor was investigated by a *t*-test at 95% confidence, and the results were indicated by the *p*-value, with *p*-values less than 0.05 showing a significant influence on the response. To avoid overfitting in the regression models, the reduced models were constructed using only the significant factors. As presented in Table 6, the reduced regression equations demonstrate higher predicted R-squared values, signifying the generalization capability and improved representation of the data relationships.

**Figure 8:**
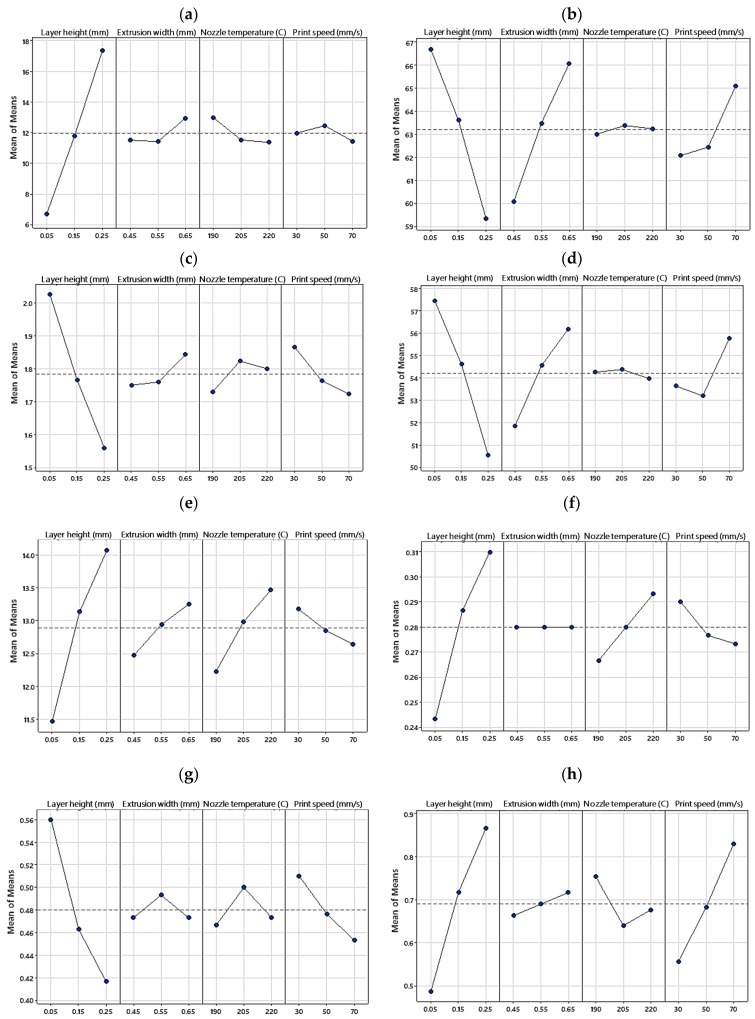
Main effects plot for means of (**a**) surface roughness, (**b**) compressive strength, (**c**) compressive modulus, (**d**) specific strength, (**e**) toughness, (**f**) failure strain, (**g**) hysteresis loss, and (**h**) residual strain.

**Figure 9:**
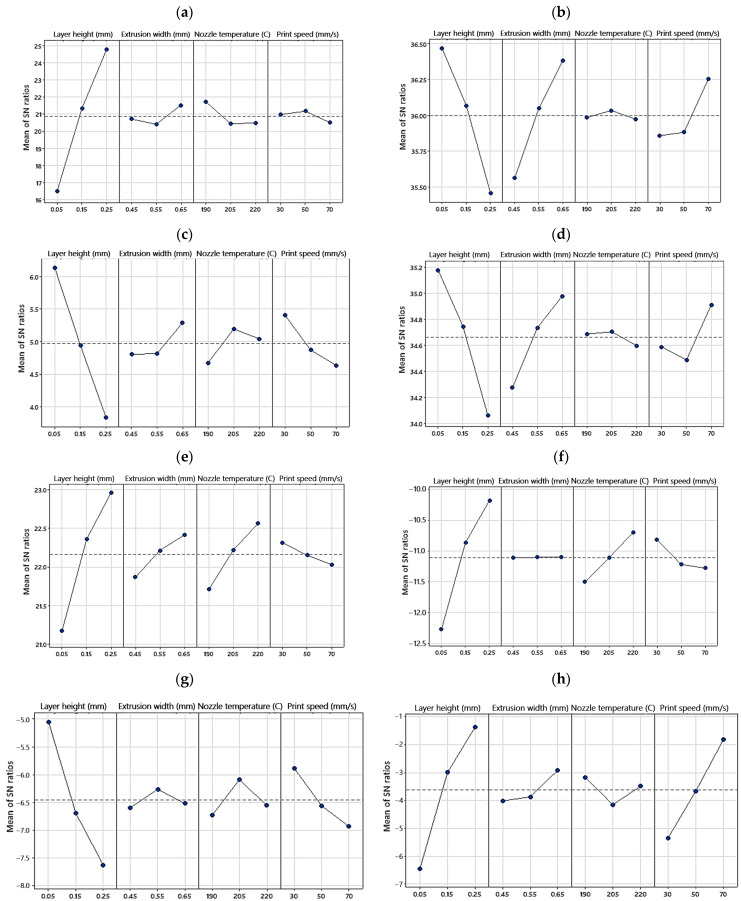
Main effects plot for S/N ratios of (**a**) surface roughness, (**b**) compressive strength, (**c**) compressive modulus, (**d**) specific strength, (**e**) toughness, (**f**) failure strain, (**g**) hysteresis loss, and (**h**) residual strain.

To gain a better understanding of the influence of each factor, the signal-to-noise (S/N) ratio was calculated with the help of Equation (5).

(5) $$S/N=-10\log_{10}\left[\frac{1}{n}\sum_{i=1}^{n}\frac{1}{{y_{i}}^{2}}\right]$$

where n is the total number of experiments, and y_i_ is the response value for the i_th_ experiment. A higher S/N ratio indicates that the factor has a stronger influence on the response variable. Figure 8 and Figure 9 illustrate the main effect plots of the means and S/N ratios for each response, respectively. “Main effect” refers to the impact of a single factor on the response variable, irrespective of the influence of other factors. The main effect of the means (or S/N ratios) for factor x is calculated by subtracting the overall mean of y (or S/N ratios) from the mean of y (or S/N ratios) at each level of x.

From Figure 8 it can be seen that changing the layer height leads to the highest variation in all the responses as further supported by Figure 9, which displays the magnitude of the S/N ratios for each factor. Figure 9 demonstrates that for all the investigated responses, the S/N ratio is maximized at the 0.05 mm layer height, while the minimal S/N value is attained at 0.25 mm, suggesting that the layer height has a predominant influence on the studied properties.

Figure 8 also provides correlations between the responses and factors. While the surface roughness, failure strain, toughness, and residual strain show a positive correlation with the layer height, the compressive strength, compressive modulus, specific strength, and hysteresis loss exhibit a negative correlation. The layer height was found to have the most significant influence on all the studied properties, followed by the print speed (for compressive modulus, hysteresis loss, and residual strain), extrusion width (for compressive strength and specific strength), and nozzle temperature (for toughness and failure strain) as the second most influential factor.

In the following, the impact of the process parameters on the studied responses will be discussed in detail.

### 3.2. Relative Density

**Figure 10:**
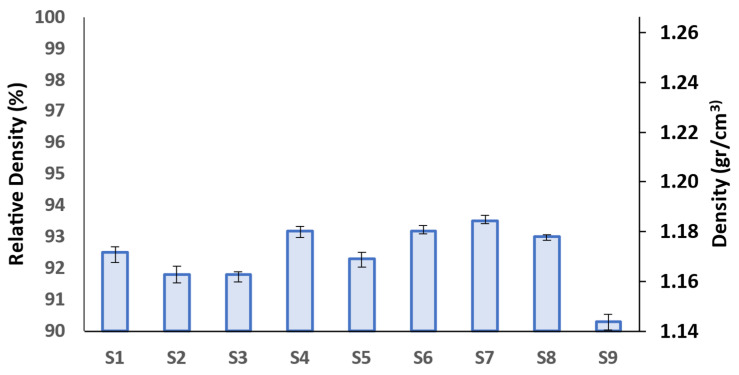
Relative density of 3D-printed PLA specimens (error bars: standard deviations).

The density of the 3D-printed specimens is depicted in Figure 10.

As can be seen in Figure 10, the relative density of the samples is almost identical, ranging between 90.3% and 93.5%. This shows that altering the design parameters does not significantly affect the density of the samples. The remaining porosity within the samples is attributed to the incomplete bonding between the layers as well as the round shape of the extruded filaments as reported elsewhere [58,59]. Nevertheless, some reports suggest that increasing the nozzle temperature [60] and reducing the print speed [61] can greatly enhance the relative density of FFF prints.

### 3.3. Surface Roughness

**Figure 11:**
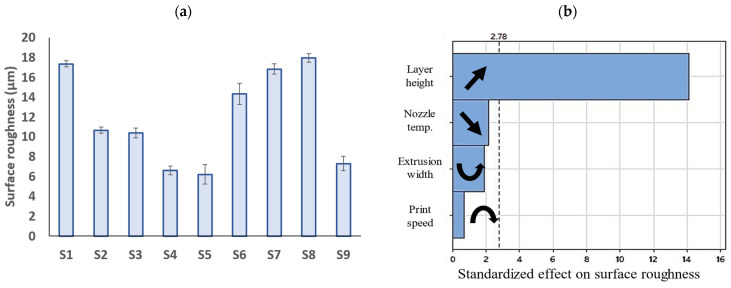
(**a**) Surface roughness of the fabricated PLA prints (error bars: standard deviations), and (**b**) Pareto charts of standardized effects of 3D printing parameters on surface roughness (black arrows in the figure indicate the correlation/trend between the process parameters and the response).

Figure 11a illustrates the surface roughness of the PLA prints. As can be seen, the surface texture can vary greatly depending on the process parameters, with the S5 and S8 samples having the lowest (~6µm) and the highest (~18µm) surface roughness, respectively. To simplify the significance of each factor and its correlation to the surface roughness, the Pareto chart of the standardized effects is shown in Figure 11b. In this chart, the factors were ranked in order of their impact on the properties studied. Standardized effects larger than the vertical dotted line show a significant effect on the response, with the factors having a higher standardized effect showing a higher influence. The black arrows indicate the correlation/trend of each factor with the response.

**Figure 12:**
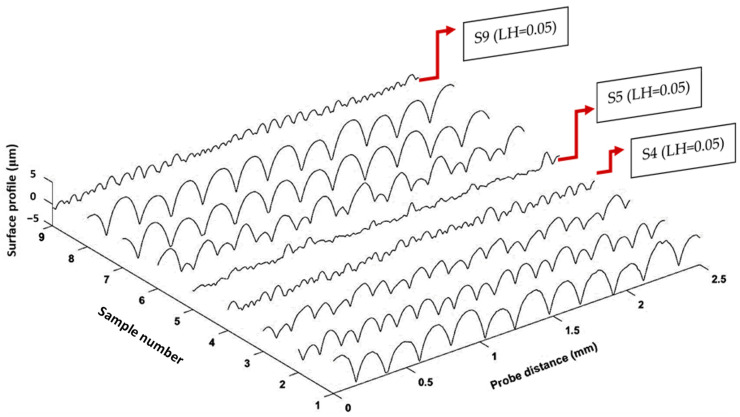
Surface profile of FFF samples along the height direction.

It has been shown that parameters such as layer height, extrusion width, raster angle, and nozzle temperature are influential on the surface quality of FFF prints [62]. In the present study, the layer height was identified as the only influential factor on the surface quality of the prints and other parameters, i.e., the extrusion width, print speed, and nozzle temperature exhibited a nonsignificant impact on the results as demonstrated in Figure 11b. In the literature, the layer height was shown to be the most influential factor among other parameters when the surface quality is measured in the height direction [17,43]. This is because the surface roughness is highly affected by the peaks and valleys between the deposited layers in a print, as depicted by Z(x) in Figure 6. According to Equation (1), decreasing the layer height reduces the Z distance and thus decreases the surface roughness. Figure 12 illustrates the surface profiles of the FFF samples along the height dimension. As can be seen, the samples with the lowest layer height (S4, S5, and S9) exhibit the smoothest surfaces with the minimal distance between the peaks and valleys.

A similar result was reported in [46] where the extrusion width was identified as the most influential factor in the surface roughness of the PLA prints, while the nozzle temperature showed little influence. In the mentioned study, the surface roughness was measured at the top surface of the prints where the stylus sweeps the extruded filaments from the width direction.

### 3.4. Compressive Properties

**Figure 13:**
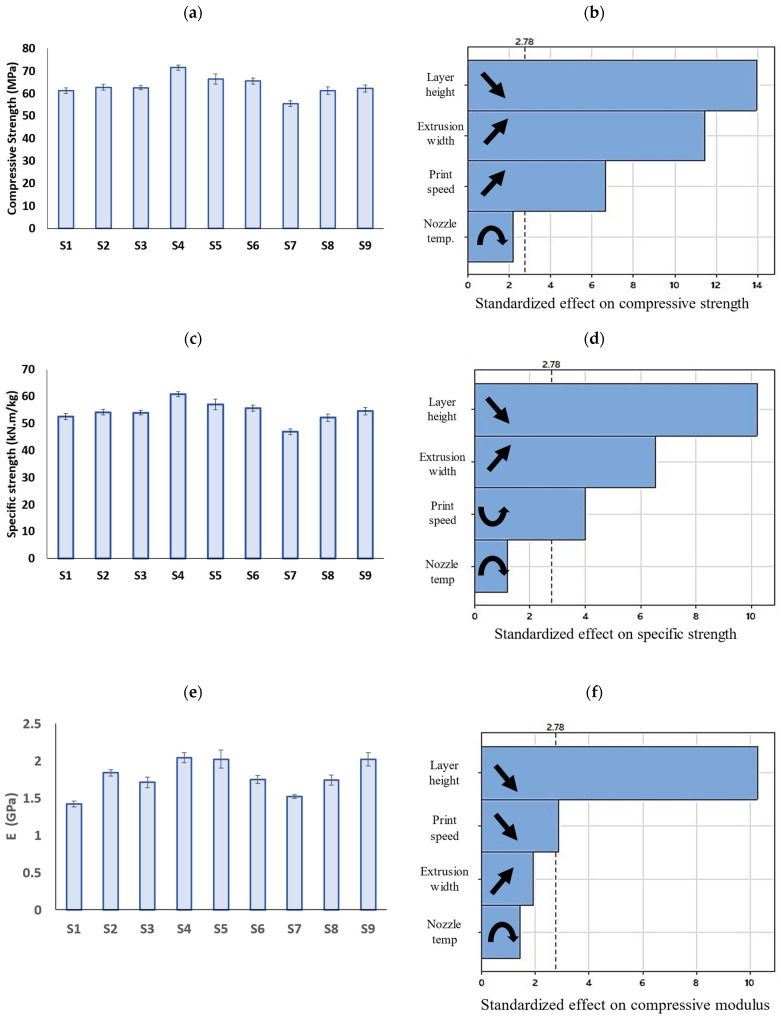
Bar graphs of (**a**) compressive strength, (**c**) specific strength, and (**e**) compressive modulus of PLA prints (error bars: standard deviations) and (**b**,**d**,**f**) their corresponding Pareto charts.

Figure 13 demonstrates the compressive properties extracted from the compression tests as well as the Pareto charts for each property. As can be seen, the compressive modulus, compressive strength, and specific strength fall within the range of 1.42–2.04 GPa, 55.5–71.5 MPa, and 46.96–60.79 kN.m/kg, respectively. The S4 sample exhibited the highest compressive strength, compressive modulus, and specific strength among the samples.

**Figure 14:**
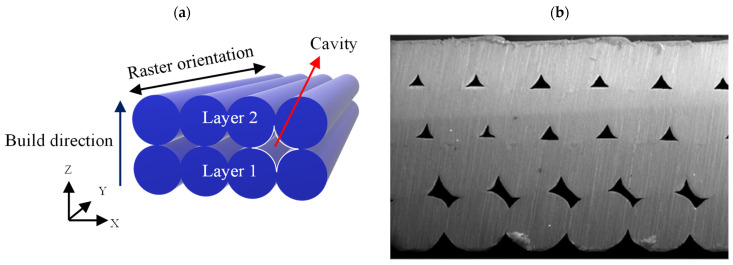
(**a**) Anisotropy of FFF prints caused by build orientation, raster orientation, and cavities, and (**b**) internal structure of an ABS print [64] (CC BY 4.0).

According to the Pareto charts in Figure 13, the compressive strength, modulus, and specific strength are inversely proportional to the layer height. However, there has been contradicting reports in the literature, exhibiting direct [30], inverse [35], and insignificant [40] effects of the layer height on the compressive strength of FFF prints. Wu et al. [40] reported that while the thickness of the layers greatly affected the tensile strength, it had minimal impact on the bending and compressive strengths of PEEK prints. Sood et al. [30] examined the effect of different layer heights (0.127, 0.178, and 0.254 mm) on the compressive strength of ABS prints and found that a decrease in the layer height causes a decrease in compressive stress. They concluded that reducing the layer height results in a higher number of layers and this leads to higher distortions arising from the thermal stress accumulating between the layers. Conversely, Nomani et al. [35] achieved contradicting results which are in agreement with the present study. Based on their results, the compression testing of ABS prints at different layer heights ranging from 0.2 mm to 0.8 mm revealed samples printed at the smallest examined layer height of 0.2 mm attained the highest compressive strength and modulus. Their results showed that a higher layer height led to a greater residual porosity and lower hardness in the bulk material, which could explain the observed decrease in mechanical strength. In fact, due to the circular cross section of the filaments, some internal cavities are formed between the rasters (Figure 14), resulting in anisotropic properties and decreased strength in FFF prints under compression [58,63]. Reducing the layer height leads to smaller internal cavities (porosity) between layers, resulting in enhanced mechanical properties. A porosity increase with the layer height has been reported in the literature [27,59]. Furthermore, decreasing the layer height has been shown to increase the contact area between extruded filaments. This increases the heat transmission between layers and promotes layer adhesion [26].

The results obtained by the ANOVA analysis also revealed a direct relationship between the compressive properties of the PLA prints and the extrusion width. While it has been reported that the extrusion width has an insignificant impact on the compressive strength of ABS prints [14], the results of the present study, in Figure 8b–d, showed that altering the extrusion width from 0.45 mm to 0.65 mm led to an increase in the compressive strength and specific strength by 10% and 8.5%, respectively. However, the impact of the extrusion width on the compressive modulus was not significant. Altering the extrusion width can affect the compressive strength of FFF prints as it influences the bonding characteristics between layers. By increasing the extrusion width, the contact area between layers is increased, leading to stronger bonding and greater strength in the printed part.

Finally, the print speed exhibited a different impact on the compressive properties of the PLA prints. While increasing the print speed resulted in a higher compressive strength, it had an inverse effect on the compressive modulus. When polymers are printed at lower speeds, the filaments remain in contact with the heated nozzle for a longer period of time, increasing the temperature within the filament. High-temperature exposure affects the crystallization of PLA and induces thermal degradation, resulting in a loss in mechanical characteristics [65]. In addition, at lower print speeds, the time between depositing a layer and the hot nozzle touching it again to deposit another layer on top (known as the thermal cycle) increases, leading to a higher temperature gradient between adjacent layers. Because the preceding layer has undergone significant cooling, the overall energy of the polymer at the interface may fall short of facilitating adequate molecular chain fusion and coalescence, ultimately leading to suboptimal interlayer bonding. This results in weaker bonds and lower compressive strength. If the temperature between these layers is insufficient, it can hinder molecular chain fusion, resulting in weak interlayer bonding. Higher print speeds reduce the time between layers, minimizing cooling and maintaining a higher temperature, and thus enhancing mechanical performance. Zhang et al. [66] predicted a positive link between the print speed and FDM component strength due to better thermal coalescence. Likewise, Samy et al. [66] found that higher nozzle speeds in FFF printing prevented significant cooling between layers, reducing the residual stress and strengthening the bonds. However, there are some reports suggesting that print speed has no significant effect on compressive strength [15,21].

**Figure 15:**
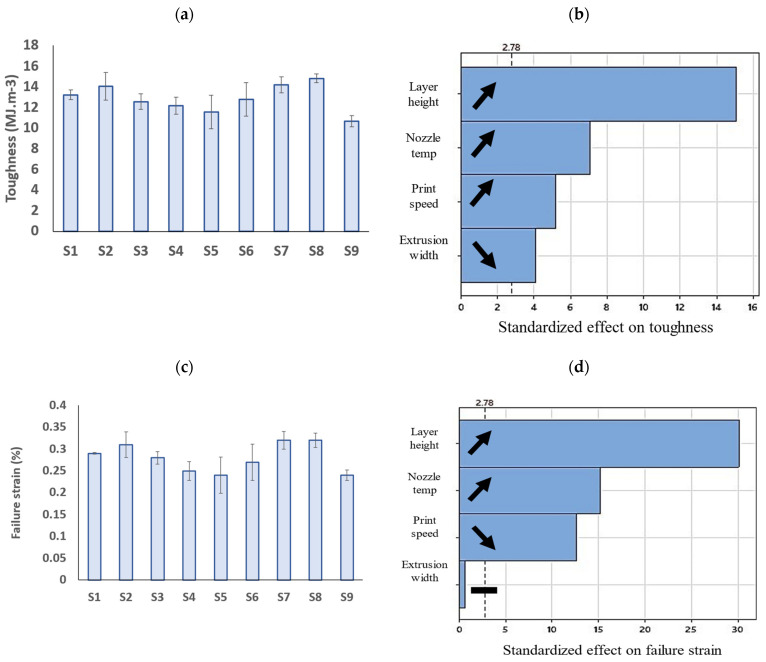
Bar graphs of (**a**) toughness and (**c**) failure strain of PLA prints (error bars: standard deviations) and (**b**,**d**) their corresponding Pareto charts.

As shown in Figure 15, the layer height and nozzle temperature exhibited the highest impact with a direct correlation to the toughness and failure strain of the PLA prints. Although the data on the compressive toughness and failure strain of FFF prints are lacking in the literature, some data show that these properties in tensile loading are not remarkably affected by 3D printing parameters [67]. It is believed that when the layer height is increased, each layer has more material deposited, resulting in a larger surface area for the next layer to bond to. Similarly, when the nozzle temperature is increased, the PLA material is heated to a higher temperature, which makes it more malleable and easier to bond to adjacent layers. Enhanced interlayer bonding leads to a higher toughness in PLA prints, as the layers are less likely to separate under stress or impact. The print speed was also shown to have a direct correlation with the toughness and an inverse relation with the failure strain.

### 3.5. Hysteresis Properties

**Figure 16:**
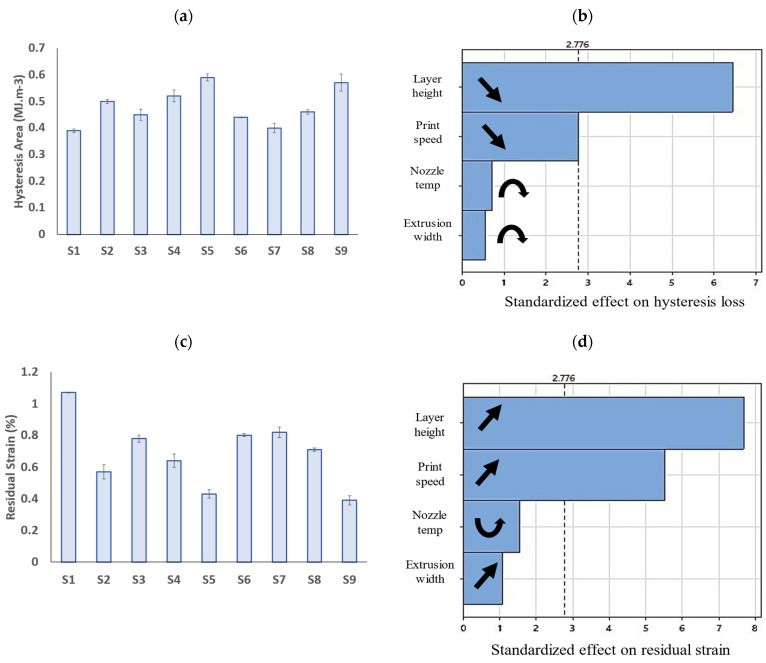
Bar graphs of (**a**) hysteresis area (or hysteresis loss) and (**c**) residual strain of PLA prints (error bars: standard deviations) and (**b**,**d**) their corresponding Pareto charts.

Figure 16 illustrates the hysteresis loss and residual strains extracted from the hysteresis curves of the PLA prints. The S5 sample exhibited the biggest hysteresis loss, showing a higher damping capacity than the other samples. The S9 sample also had the lowest residual strain after unloading.

According to the Pareto charts in Figure 16b,d, the hysteresis properties of the PLA prints are affected the most by the layer height, followed by the print speed, while the nozzle temperature and extrusion width showed a little impact on the results. The layer height and print speed exhibited a direct correlation with the residual strain and an inverse relation with the hysteresis loss. That is, by decreasing the layer height or print speed, the residual strain of a PLA print decreases while its hysteresis loss increases, which is a favorable outcome. Because the compression hysteresis of FFF prints has not been studied thoroughly in the literature, the underlying mechanism of these observations is not fully understood. It is believed that by decreasing the layer height, the porosity of the structure will decrease, resulting in a higher hysteresis loss. The direct relation between the density of polymers and their hysteresis loss has been reported for polymeric foams [68] and FFF prints [56]. In addition, samples with a higher relative density possess more rigid structures which allow them to recover their shape after unloading, resulting in lower residual strain. Decreasing the print speed has also resulted in an increase in the hysteresis loss. This is mainly due to the higher residual stresses within the prints induced by higher magnitudes of temperature gradients at low printing speeds. It has been shown that the induction of compressive residual stresses can increase the damping capacity of materials [69].

### 3.6. Optimization

Two optimization schemes were followed in this study. In the first scheme (A), the goal was to achieve the highest compressive strength, regardless of the other characteristics. In the second scheme (B), the optimization problem was solved to maximize the compressive strength, compressive modulus, specific strength, and toughness while minimizing the surface roughness. The optimization goals for each scheme were chosen based on the potential industrial and biomedical applications of PLA prints. For example, in plastic enclosures for electronic equipment, plastic tools handles, automotive interior components, or some surgical instruments, the main focus is on the static strength of the prints. However, in various biomedical applications such as bone scaffolds [70], a combination of factors including the mechanical strength, compressive modulus, toughness, and surface roughness of the prints are all taken into consideration.

Table 7 provides five candidate designs for each scheme with the predicted responses and their corresponding desirability index (DI). Letters A and B denote the optimum designs for schemes A and B, respectively. The first two optimum designs with the highest desirability index were chosen for experimental validation, making a total of four designs (A1, A2, B1, and B2). To validate the optimization results, three samples were fabricated for each design and then were subjected to mechanical and physical tests as described earlier. As can be seen in Table 7, A2 and B1 represent the same design, implying that this design is optimum for both schemes. In addition, when only the highest compressive strength is desired (scheme A), the first optimum design (A1) is the same as sample S4 which was fabricated and tested earlier. So, there was no need to test the A1 sample again as its properties already existed. As shown in Table 8, the average of the measured values for each characteristic was computed and compared to the corresponding predicted values. The A1 design possesses the highest compressive strength (71.49 MPa) followed by A2 and B2, respectively. The highest performance was achieved in the B1 (=A2) sample when the compressive properties and surface quality are desired. Compared to B2, the B1 sample showed a higher compressive strength, compressive modulus, failure strain, toughness, and surface quality, while the B2 sample marginally possessed a higher relative density, specific strength, and hysteresis loss.

**Figure 17:**
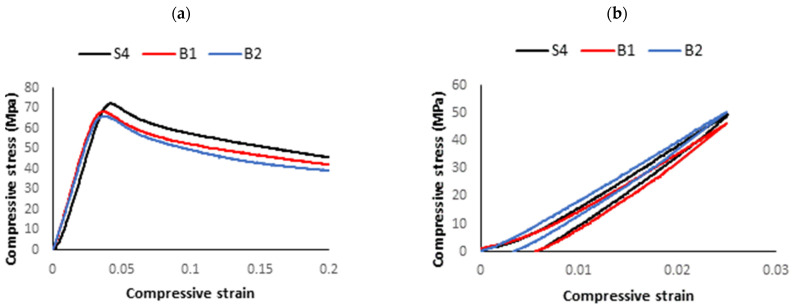
(**a**) Compressive stress–strain and (**b**) hysteresis–compression curves of optimum samples.

Figure 17 shows the compressive stress–strain and hysteresis curves of the optimum samples.

### 3.7. Failure Analysis

**Figure 18:**
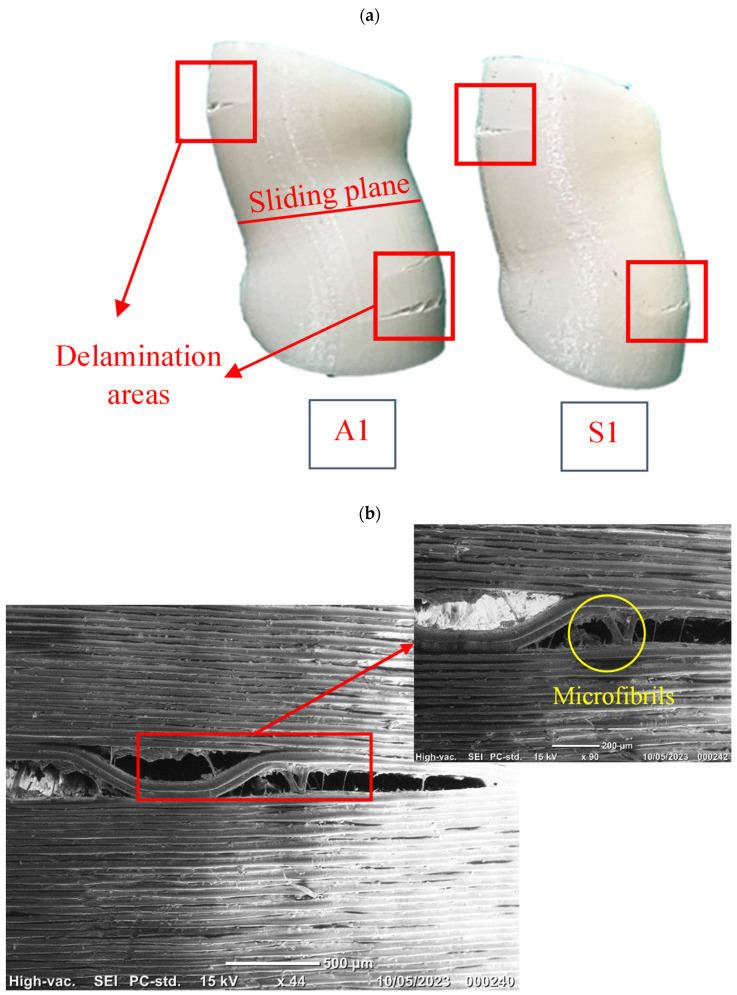

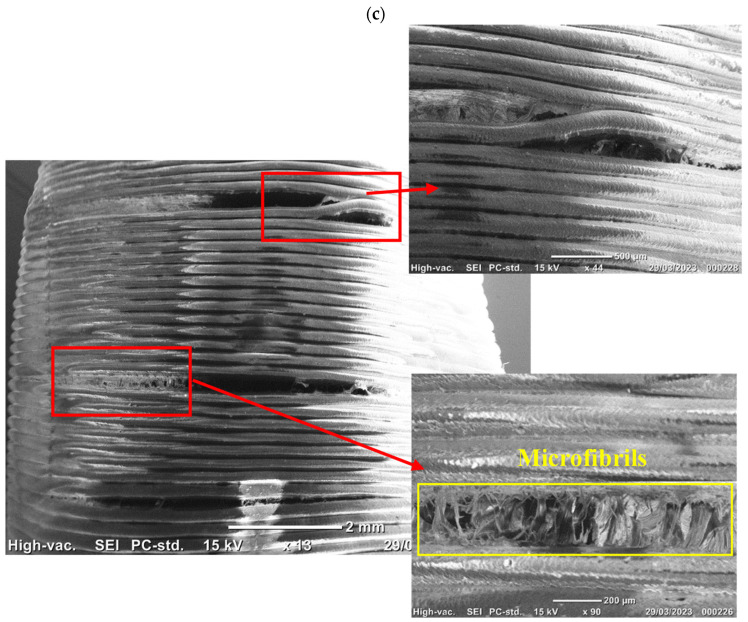
(**a**) Sliding and delamination of layers under compression, and SEM images of the delamination area in samples (**b**) A1 and (**c**) S1.

A failure analysis was performed visually and using scanning electron microscopy. The observations clearly showed that the main failure mechanism in all the tested samples was sliding the layers in the middle section of the specimens. As evidenced in Figure 18a, further compressing the samples resulted in a rupture caused by the delamination between the layers. The same observation has been reported in the literature for FFF prints under compression [30,71]. The SEM images revealed that delamination occurs at the highly stressed points along the height of the samples. In Figure 18b,c, the failure images of the A1 and S1 samples are shown in two different magnifications, highlighting the delamination characteristics at the interfaces. Clean delamination suggests the possible existence of defects or the lack of interlayer adhesion as the layers were deposited. In contrast, microfibrils suggest strong interlayer bonding due to a high degree of fusion between the layers upon deposition, which is similar to cohesive failures in the fibers of perfectly fused (bonded) layers. The symmetry of these fractures was also noteworthy in the observations. It is believed that the interlaminar shearing, which seems to primarily drive the failure of the core, caused a stress concentration symmetrically around the axis. A stress concentration drives the existing defects into debonding the printed layers, resulting in the fracture of specimens.

**Figure 19:**
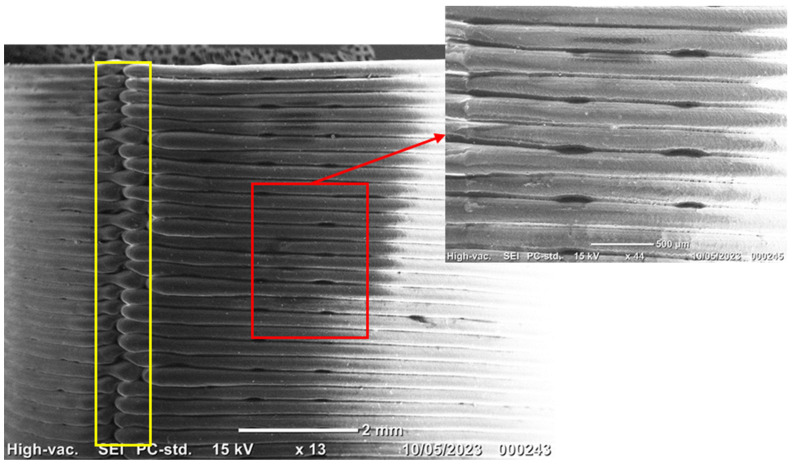
Gaps and discontinuities at the interface areas (red box) and stich line (yellow box) in as-fabricated S1 sample.

One common observation among all the samples that has received less attention in the literature is that all the delamination points were close to the so-called stitch line, as depicted by the yellow box in Figure 19. As shown in this figure, the bulged area in each layer is the starting point where the hot nozzle starts depositing the filament and finishes the layer at the same point. The stich line is a line connecting the starting points of the layers. The shape discontinuity and gaps in the stich line cause the stress concentration. The proximity of the interlayer gaps (as shown by the red box) to the stitch line has resulted in the failures starting in this stress-concentrated region. One solution to this might be changing the starting point in each layer, using a different path planning strategy.

## 4. Conclusions

In this study, the effect of the layer height, extrusion width, nozzle temperature, and print speed on the compressive and surface properties of PLA specimens was investigated. The experiments were designed using the orthogonal array Taguchi method, and the 3D-printed specimens were subjected to uniaxial compression and hysteresis compression testing. The relative density and surface roughness of the specimens were also extracted. The most significant impact on the studied properties was found to be caused by changes in the layer height, which resulted in the highest variation in all the responses. Reducing the layer height was found to increase the compressive strength, compressive modulus, and hysteresis loss while reducing the surface roughness and residual strain. However, a decrease in the toughness and failure strain was observed for the PLA prints with a reduced layer height. Additionally, print speed was identified as the second most influential factor affecting the compressive modulus, hysteresis loss, and residual strain. The relative density of the specimens was found to be insensitive to the studied parameters, ranging from 90.3% to 93.5%. According to the optimization results, the highest compressive strength was obtained at a layer height of 0.05, an extrusion width of 0.65, a nozzle temperature of 220 °C, and a print speed of 70 mm/s. The failure analysis revealed that interlayer sliding and layer debonding were the main failure mechanisms for the PLA prints under compression. It was also noted that the interlayer gaps and shape discontinuity in the stich line in the FFF prints caused the formation of highly stress-concentrated areas, resulting in the subsequent layer debonding and rupture of the specimens.

One potential avenue for future research is the exploration of post-processing techniques to enhance print quality and compressive properties. Investigating various methods such as thermal and chemical treatments can yield valuable insights into improving the final product. Furthermore, it would be valuable to delve deeper into the impact of environmental factors, such as temperature and humidity, on the compressive properties of 3D prints. A comprehensive study could help establish guidelines for better design practices, including optimal printing conditions under different environmental conditions. Another area ripe for exploration is the detailed investigation on the effect of process parameters on the anisotropic behavior of prints under compression. By fine-tuning these parameters, researchers can optimize FFF settings for specific applications, potentially expanding the range of industries where FFF printing can be effectively utilized. Lastly, understanding the intricate relationship between the 3D printing parameters and microstructural properties of produced parts is of paramount importance for enhancing the compressive strength of prints. A thorough analysis in this regard can lead to innovative solutions for creating stronger and more resilient components.

## Figures and Tables

**Table 2:** Variables, responses, and levels specified for the design of experiments.

Variables	Unit	Low (−1)	Medium (0)	High (+1)
Layer height (LH)	mm	0.05	0.15	0.25
Extrusion width (EW)	mm	0.45	0.55	0.65
Nozzle temperature (T)	°C	190	205	220
Print speed (V)	mm/s	30	50	70
**Responses**				
Relative density	$\rho_{rel}=\rho_{sample}/\rho_{Filament}\times 100$			
Surface roughness	$R_{a}=\int_{0}^{l}\left|Z(x)\right|dx/l$			
Compressive strength	$\sigma_{y}=0.2\%proofstrength$			
Compressive modulus	E=Slopeofthelinearpartof(σ−ε)curve			
Specific strength	$\sigma_{s}=\sigma_{y}/\rho_{sample}$			
Failure strain	$\varepsilon_{f}=Strainatfailurepoint$			
Hysteresis loss (Area)	$A_{hys}=Areaconfinedbytheloading-unloadingcurves$			
Residual strain	$\varepsilon_{r}=Residualstrainafterunloading$			

**Table 3:** Design of experiment (orthogonal array Taguchi method).

Sample No.	Layer Height (mm)	Extrusion Width (mm)	Nozzle Temperature (°C)	Print Speed (mm/s)
S1	0.25	0.55	190	70
S2	0.15	0.55	220	30
S3	0.15	0.45	205	70
S4	0.05	0.65	220	70
S5	0.05	0.55	205	50
S6	0.15	0.65	190	50
S7	0.25	0.45	220	50
S8	0.25	0.65	205	30
S9	0.05	0.45	190	30

**Table 4:** Compressive properties and surface roughness of the PLA prints.

No.	T (min)	$\rho_{rel}\;$ (%)	Ra (µm)	$\sigma_{max}$(MPa)	E (GPa)	σ/*ρ* (kN.m/kg)	$T_{C}$ $\left(MJ.m^{-3}\right)$	$\varepsilon_{f}\;$ (%)	$A_{Hys}$ $\;(MJ.m^{-3})$	$\varepsilon_{r}\;$ (%)
S1	14	92.50	17.35	61.30	1.42	52.53	13.21	0.29	0.39	1.07
S2	32	91.80	10.67	62.78	1.84	54.17	14.05	0.31	0.50	0.57
S3	25	91.80	10.40	62.55	1.71	54.01	12.56	0.28	0.45	0.78
S4	60	93.20	6.59	71.49	2.04	60.79	12.15	0.25	0.52	0.64
S5	70	92.30	6.22	66.38	2.02	56.98	11.57	0.24	0.59	0.43
S6	23	93.20	14.32	65.50	1.75	55.65	12.79	0.27	0.44	0.80
S7	17	93.50	16.83	55.46	1.52	46.96	14.20	0.32	0.40	0.82
S8	18	93.00	17.95	61.22	1.74	52.15	14.81	0.32	0.46	0.71
S9	106	90.30	7.30	62.23	2.02	54.59	10.68	0.24	0.57	0.39

**Table 5:** Analysis of variance (results) *.

	DF	Adj SS	Adj MS	F-Value	*p*-Value	Remarks
**Surface roughness**	$={\left(2.208+7.903LH\right)}^{2}$					
Regression	4.000	178.390	44.598	52.060	0.001	Significant
Layer height (mm)	1.000	170.880	170.880	199.480	<0.001	Significant
Extrusion width (mm)	1.000	3.125	3.125	3.650	0.129	Insignificant
Nozzle temperature (C)	1.000	3.969	3.969	4.630	0.098	Insignificant
Print speed (mm/s)	1.000	0.416	0.416	0.490	0.524	Insignificant
Error	4.000	3.426	0.857			
Total	8.000	181.816				
**Compressive strength**	$=109.25\;\sqrt{5.92-37.49\;LH+30.65\;EW}$					
Regression	4.000	167.300	41.825	94.000	<0.001	Significant
Layer height (mm)	1.000	86.762	86.762	195.000	<0.001	Significant
Extrusion width (mm)	1.000	58.500	58.500	131.480	<0.001	Significant
Nozzle temperature (C)	1.000	2.134	2.134	4.800	0.094	Insignificant
Print speed (mm/s)	1.000	19.904	19.904	44.730	0.003	Significant
Error	4.000	1.780	0.445			
Total	8.000	169.079				
**Compressive modulus**	$=3.07\times \sqrt[{3}]{1.0252-2.359\;LH-0.003\;V}$					
Regression	4.000	4.664	1.166	29.950	0.003	Significant
Layer height (mm)	1.000	4.114	4.114	105.650	0.001	Significant
Extrusion width (mm)	1.000	0.146	0.146	3.760	0.125	Insignificant
Nozzle temperature (C)	1.000	0.081	0.081	2.090	0.222	Insignificant
Print speed (mm/s)	1.000	0.323	0.323	8.290	0.045	Significant
Error	4.000	0.156	0.039			
Total	8.000	4.819				
**Specific strength**	$=85.85\sqrt[{3}]{3.83-34.77\;LH+22.1\;EW+0.064\;V}$					
Regression	4.000	116.649	29.162	41.060	0.002	Significant
Layer height (mm)	1.000	73.994	73.994	104.170	0.001	Significant
Extrusion width (mm)	1.000	30.294	30.294	42.650	0.003	Significant
Nozzle temperature (C)	1.000	1.032	1.032	1.450	0.295	Insignificant
Print speed (mm/s)	1.000	11.330	11.330	15.950	0.016	Significant
Error	4.000	2.841	0.710			
Total	8.000	119.491				
**Toughness**	$=20.36\times \sqrt[{3}]{-7.76+13.122\;LH+3.573\;EW+0.04104\;T-0.02265\;V}$					
Regression	4.000	14.602	3.651	80.440	<0.001	Significant
Layer height (mm)	1.000	10.331	10.331	227.640	<0.001	Significant
Extrusion width (mm)	1.000	0.766	0.766	16.880	0.015	Significant
Nozzle temperature (C)	1.000	2.274	2.274	50.100	0.002	Significant
Print speed (mm/s)	1.000	1.231	1.231	27.130	0.006	Significant
Error	4.000	0.182	0.045			
Total	8.000	14.784				
**Failure strain**	$=0.385\times \sqrt[{6}]{-307.54+0.3621\;LH+0.001218\;T-0.000758\;V}$					
Regression	4.000	0.011	0.003	325.020	<0.001	Significant
Layer height (mm)	1.000	0.008	0.008	908.720	<0.001	Significant
Extrusion width (mm)	1.000	0.000	0.000	0.420	0.553	Insignificant
Nozzle temperature (C)	1.000	0.002	0.002	231.480	<0.001	Significant
Print speed (mm/s)	1.000	0.001	0.001	159.450	<0.001	Significant
Error	4.000	0.000	0.000			
Total	8.000	0.011				
**Hysteresis loss**	$={\left(3.109\;LH+0.00651\;V-1.331\right)}^{-1}$					
Regression	4.000	12.544	3.136	12.540	0.016	Significant
Layer height (mm)	1.000	10.406	10.406	41.610	0.003	Significant
Extrusion width (mm)	1.000	0.076	0.076	0.310	0.610	Insignificant
Nozzle temperature (C)	1.000	0.126	0.126	0.500	0.517	Insignificant
Print speed (mm/s)	1.000	1.935	1.935	7.740	0.050	Significant
Error	4.000	1.001	0.250			
Total	8.000	13.544				
**Residual strain**	$={\left(0.4397+1.171\;LH+0.004129\;V\right)}^{2}$					
Regression	4.000	0.342	0.085	23.330	0.005	Significant
Layer height (mm)	1.000	0.217	0.217	59.140	0.002	Significant
Extrusion width (mm)	1.000	0.004	0.004	1.160	0.341	Insignificant
Nozzle temperature (C)	1.000	0.009	0.009	2.410	0.196	Insignificant
Print speed (mm/s)	1.000	0.112	0.112	30.600	0.005	Significant
Error	4.000	0.015	0.004			
Total	8.000	0.356				

* DF: degrees of freedom; Adj SS: adjusted sum of squares; Adj MS: Adj SS/DF; F-value: the ratio of the mean square for each factor to the mean square for error; *p*-value: the probability of obtaining a test statistic as extreme or more extreme than the observed value, assuming the null hypothesis is true.

**Table 6:** Predictive performance of regression models.

	Predicted R-sq (%) (Full Model)	Predicted R-sq (%) (Reduced Model)	Improvement (%)
Surface roughness	89.28	92.15	3.21
Compressive strength	93.53	94.5	1.04
Compressive modulus	82.25	83.24	1.2
Specific strength	84.87	91.54	7.86
Failure strain	98.53	99.05	0.53
Hysteresis loss	64.48	81.53	26.44
Residual strain	82.71	87.66	5.98

**Table 7:** Optimum designs for schemes A and B (selected designs with the highest DI indexes are shown in bold format).

No.	LH	EW	T	V	$\rho_{rel}$	Ra	$\sigma_{max}$	E	σ/ρ	$T_{C}$	$\varepsilon_{f}$	$A_{Hys}$	$\varepsilon_{r\;}(\%)$	DI
**A1**	**0.05**	**0.65**	**220**	**70**	**92.47**	**6.77**	**71.46**	**2.02**	**61.02**	**12.28**	**0.25**	**0.52**	**0.61**	**1.00**
**A2**	**0.05**	**0.65**	**205**	**70**	**92.01**	**6.66**	**71.46**	**2.02**	**61.02**	**11.82**	**0.24**	**0.55**	**0.58**	**1.00**
A3	0.05	0.65	190	70	91.63	7.45	71.46	2.02	61.02	11.20	0.22	0.51	0.69	0.97
A4	0.05	0.55	190	70	91.63	6.86	68.88	1.98	59.38	10.98	0.22	0.51	0.69	0.84
A5	0.05	0.55	220	70	92.47	6.28	68.88	1.98	59.38	12.02	0.25	0.52	0.61	0.84
**B1**	**0.05**	**0.65**	**205**	**70**	**92.01**	**6.66**	**71.46**	**2.02**	**61.02**	**11.82**	**0.24**	**0.55**	**0.58**	**0.85**
**B2**	**0.05**	**0.65**	**220**	**30**	**91.66**	**6.77**	**68.42**	**2.11**	**58.87**	**12.28**	**0.26**	**0.60**	**0.34**	**0.84**
B3	0.05	0.65	220	70	92.47	6.77	71.46	2.02	61.02	12.28	0.25	0.52	0.61	0.84
B4	0.05	0.65	205	30	91.21	6.66	68.42	2.11	58.87	11.82	0.25	0.63	0.30	0.82
B5	0.05	0.65	205	50	92.51	6.66	68.79	2.05	58.44	11.82	0.24	0.58	0.43	0.81

**Table 8:** Mechanical and physical properties of optimum samples.

	$\rho_{rel}$	$\sigma_{max}$	E	σ/ρ	$A_{Hys}$	T	Ra	$\varepsilon_{f}$
**A1** (**Experimental**)	**93.2**	**71.49**	**2.04**	**60.79**	**0.52**	**12.15**	**6.59**	**0.25**
A1 (Predicted)	92.47	71.46	2.02	61.02	0.52	12.28	6.77	0.25
**A2 (=B1)** (**Experimental**)	**93.6**	**67.79**	**2.29**	**56.36**	**0.56**	**12.38**	**4.92**	**0.26**
A2 (=B1) (Predicted)	92	71.46	2.02	61.02	0.55	11.82	6.66	0.24
**B2** (**Experimental**)	**93.7**	**67.15**	**2.26**	**56.77**	**0.65**	**9.82**	**5.86**	**0.21**
B2 (Predicted)	91.66	68.42	2.11	58.87	0.6	12.28	6.77	0.26

## Data Availability

Not applicable.

## Nomenclature

**LH**	Layer height	**E**	Compressive modulus
**EW**	Extrusion width	**σ/ρ**	Specific strength
**T**	Nozzle temperature	$T_{C}$	Toughness
**V**	Print speed	$\varepsilon_{f}$	Failure strain
$\rho_{rel}$	Relative density	$A_{Hys}$	Hysteresis area (hysteresis loss)
**Ra**	Surface roughness	$\varepsilon_{r}$	Residual strain
$\sigma_{max}$	Compressive strength	**DI**	Desirability index
**PLA**	Polylactic Acid	**FFF**	Fused filament fabrication
**ANOVA**	Analysis of variance	**FDM**	Fused Deposition Modeling
**PEEK**	Polyether ether ketone	**ABS**	Acrylonitrile butadiene styrene
